# Comparison of spinal instability and postoperative complications between laminoplasty and laminectomy surgery for spinal cord tumors

**DOI:** 10.1097/MD.0000000000042236

**Published:** 2025-04-18

**Authors:** Hyun Jin Yoo, Sung Hyun Noh, Sang Hyun Kim, Pyung Goo Cho

**Affiliations:** a Department of Neurosurgery, Ajou University College of Medicine, Suwon-si, Republic of Korea.

**Keywords:** complications, kyphosis, laminectomy, laminoplasty, spinal cord tumor

## Abstract

This study aimed to compare kyphotic changes and postoperative complications between laminectomy and laminoplasty for spinal cord tumors. We retrospectively included 110 patients who underwent spinal cord tumor resection at the Ajou University Medical Center, Korea, between January 1994 and March 2022 to compare the complications and postoperative kyphotic changes between laminectomy and laminoplasty. A total of 59 and 51 patients underwent laminectomy and laminoplasty, respectively. The groups had similar demographic characteristics. Tumor locations were classified as cervical, thoracic, and lumbar lesions and were compared. We measured preoperative and postoperative radiological parameters and identified postoperative complications. In the laminoplasty group, we studied 7, 19, and 25 cases at the cervical, thoracic, and lumbar levels, respectively. In the laminectomy group, we studied 13, 31, and 15 cases at the cervical, thoracic, and lumbar levels, respectively. At the cervical level, lordosis from C2 to C7 was 8.66 ± 5.06° before and 15.86 ± 12.54° after surgery and was 10.5 ± 6.82° before and 9.16 ± 6.5° after surgery in the laminoplasty and laminectomy groups, respectively. At the thoracic level, kyphosis from T5 to T12 was 27.89 ± 9.93° before and 23.18 ± 9.10° after surgery and was 29.94 ± 9.56° before and 28.41 ± 12.58° after surgery in the laminoplasty and laminectomy groups, respectively. At the lumbar level, lordosis from L1 to S1 was 40.86 ± 14.12° before and 42.62 ± 10.39° after surgery and was 43.65 ± 8.47° before and 37.44 ± 13.32° after surgery in the laminoplasty and laminectomy groups, respectively. Postoperative complications, such as cerebrospinal fluid leakage, infection, and hematoma, were more frequent in the laminectomy group than in the laminoplasty group. Laminoplasty rather than laminectomy for spinal cord tumor surgery can reduce postoperative complications and prevent kyphosis.

## 1. Introduction

Surgical intervention is often necessary for tumor resection and spinal cord decompression in patients with spinal cord tumors. Various surgical techniques, including laminectomy and laminoplasty, have been developed and are used to treat spinal cord tumors.^[1–3]^ However, the optimal surgical approach that can achieve maximal tumor resection while preserving spinal alignment remains controversial.^[2,3]^

Laminoplasty has garnered attention as a potential alternative to laminectomy for spinal cord tumors.^[1–3]^ Laminoplasty offers several advantages, such as preserving spinal stability and reducing the risk of postoperative complications, including cerebrospinal fluid (CSF) leakage and hematoma.^[2–6]^ Although potentially advantageous, the clinical effectiveness and long-term outcomes of laminoplasty for spinal cord tumor surgery remain unexplored.^[4,6–10]^ A systematic review and meta-analysis conducted by Sun et al^[11]^ suggested no significant difference between the use of laminoplasty and laminectomy techniques in spinal cord tumor surgery. However, their laminoplasty group exhibited decreased postoperative spinal instability, as reported in 33 studies that provided data on the incidence of spinal deformity (laminoplasty group, n = 374; laminectomy group, n = 600). Specifically, they mentioned a potential postoperative increase in spinal instability among patients with tumors because of secondary muscle mass reduction and decreased bone quality resulting from adjuvant therapies, such as radiation treatments. In addition, McGirt et al^[12]^ included 238 patients with intradural spinal tumors and indicated that laminoplasty contributes toward reducing spinal instability.

Based on these findings, we aimed to determine the criteria for the surgical treatment of spinal cord tumors at a single institution. Specifically, we aimed to compare laminoplasty and laminectomy regarding spinal instability and postoperative complications. We intended to provide insights into determining the optimal surgical approach for spinal cord tumor surgery.

## 2. Materials and methods

### 2.1. Study design

#### 2.1.1. Ethics approval

All data were obtained from the patients’ medical records and a private surgical database of the neurosurgery spine section. The Institutional Review Board of Ajou University Medical Center approved the study protocol before study initiation (approval number: XX-2023-187). The Institutional Review Board waived the need for informed consent owing to the retrospective nature of the study.

### 2.2. Data collection

The data of 110 patients who underwent spinal cord tumor resection via laminectomy or laminoplasty between January 1994 and March 2022 were included. We divided the patients into 2 groups as follows: those undergoing laminectomy after spinal cord tumor resection and those undergoing laminoplasty.

A patient roster was established using specific inclusion criteria; moreover, pertinent patient details were extracted meticulously from the comprehensive medical records. This process was performed by a single researcher to ensure consistency. The inclusion criteria were as follows: (1) exclusive receipt of either laminectomy or laminoplasty, (2) complete clinical patient data, and (3) a follow-up duration of > 6 months. The exclusion criteria were as follows: (1) multiple surgical interventions, (2) incomplete clinical data, and (3) a postoperative follow-up duration of <6 months. The study period was standardized to preoperative, immediate postoperative, and 6 postoperative months. The average follow-up period after 6 postoperative months was 27.29 months, with minimum and maximum follow-up periods of 6 and 144 months, respectively.

In addition, radiographic and other relevant imaging data were obtained to supplement the medical records. These images are central to conducting a thorough analysis and organizing the dataset. The process involved meticulous scrutiny and organization of patient information, including demographic characteristics, medical histories, surgical specifics, postoperative outcomes, and complications. Subsequently, the data were coded systematically to facilitate statistical analyses and interpretations.

### 2.3. Surgical method

Five professors specializing in spinal surgery performed the surgeries at the Neurosurgery Department of our institution. Each surgeon selected the approach based on their preferences and clinical indications tailored to each patient’s clinical presentation. The decision-making process involved a comprehensive assessment of each patient’s condition, including the location and characteristics of the spinal cord tumor, degree of spinal compression, neurological deficits, and other factors. Based on their expertise and experience, the surgeons selected the most suitable surgical technique that aligned with the patients’ clinical needs and goals.

The surgical technique involved the removal of both bilateral medial laminae portions—including the spinous process—while conserving the laminar and facet joints at the tumor site, followed by tumor excision. In the subsequent laminoplasty, the laminar and spinous processes were reconnected using miniplates and screws. Conversely, laminectomy was completed without reconnecting the removed structures.

### 2.4. Radiographic assessment

**Figure 1.**
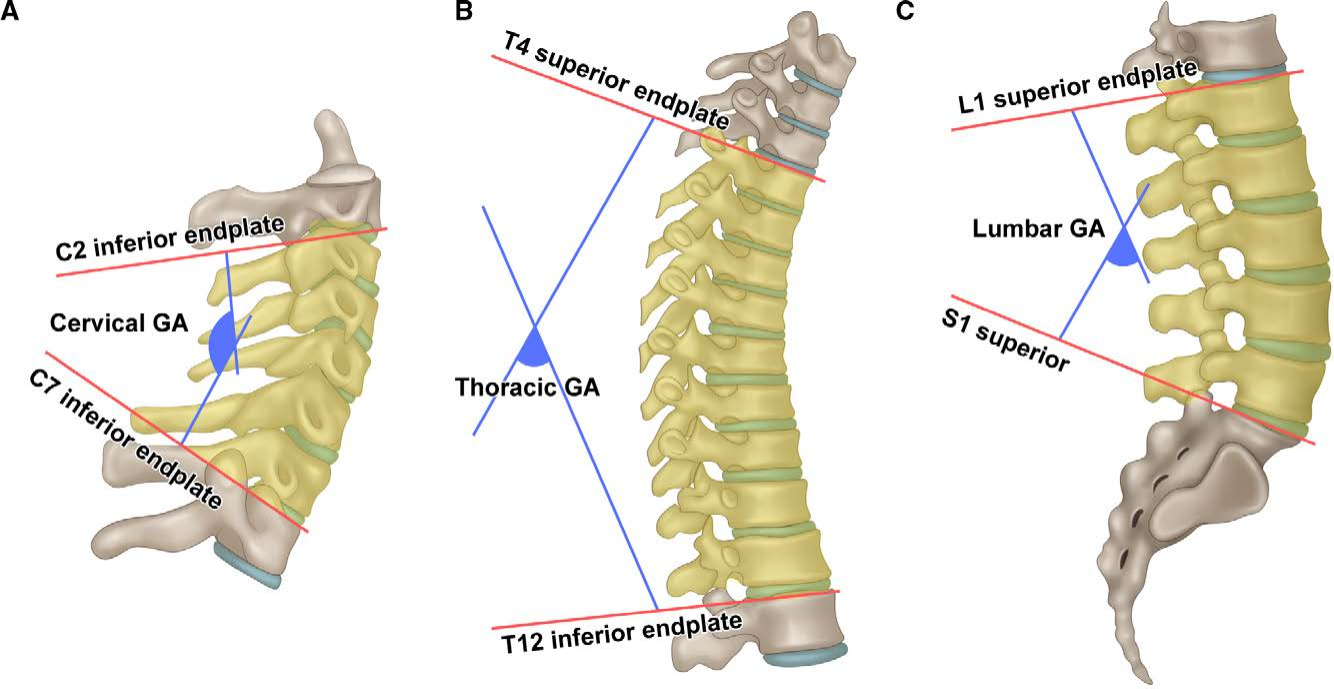
Measurement method for the global angle. At the cervical level, we measured the GA between the C2 and C7 inferior endplates. At the thoracic level, we measured the GA between the T4 superior endplate and T12 inferior endplate. Similarly, we measured the GA between the L1 and S1 superior endplates at the lumbar level.

For radiographic assessment, the “Surgi-map” software was used to register each patient’s radiographs. The global Cobb angle (GA) was measured at specific levels corresponding to the tumor location in each patient. At the cervical level, we measured GA between the C2 and C7 inferior endplates. This measurement helped us evaluate the lordotic curvature of the cervical spine. At the thoracic level, we measured the GA between the T4 superior endplate and T12 inferior endplate. This measurement allowed evaluation of the kyphotic curvature of the thoracic spine. Similarly, we measured GA between the L1 and S1 superior endplates at the lumbar level. This measurement allowed assessment of the lordotic curvature of the lumbar spine. To examine the differences in outcomes, we compared the absolute values of “GA from preoperative to immediate postoperative,” “GA from preoperative to last follow-up,” and “GA from immediate postoperative to last follow-up” (Fig. 1).

**Figure 2.**
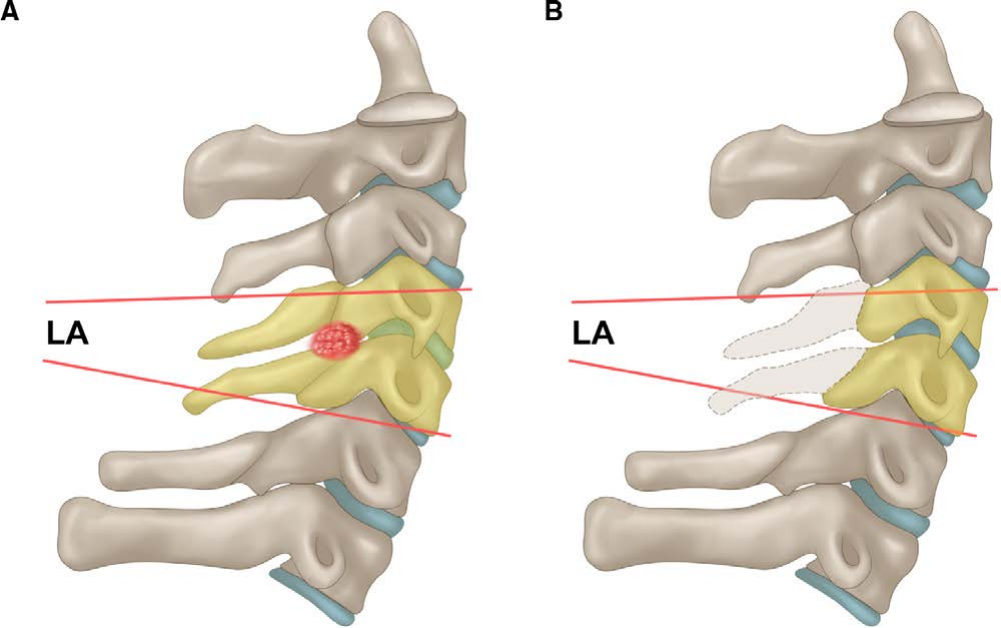
Measurement method for the local angle. This assessment involved measuring the angle between the superior endplate of the vertebra belonging to the upper tumor margin and the inferior endplate of the vertebra belonging to the lower margin.

The local Cobb angle (LA) was assessed to confirm the angle of the surgical site. This assessment involved the measurement of the angle between the superior endplate of the vertebra belonging to the upper tumor margin and inferior endplate of the vertebra belonging to the lower margin (Fig. 2). To examine outcome differences, we compared the absolute values of “LA from preoperative to immediate postoperative” and “LA from preoperative to last follow-up.”

### 2.5. Postoperative complications

Postoperative complications included CSF leakage, spinal adhesion, infection, and hematoma. In cases of CSF leakage, infection, and hematoma, we performed additional imaging tests for symptomatic patients. CSF leakage was identified based on the characteristics of the postoperative draining fluid and by observing the wound discharge. Infections were detected by monitoring postoperative body temperature and laboratory results. Hematomas were identified by assessing the patients for neurological deterioration or by imaging, such as magnetic resonance imaging. Spinal adhesions were confirmed during the follow-up magnetic resonance imaging or surgical revision in the operating room.

### 2.6. Statistical analyses

All statistical analyses were conducted using R software, version 4.0.5. Statistical significance was set at *P* < .05. The Mann–Whitney U test was performed as a nonparametric method for comparing continuous variables, whereas the chi-square test or Fisher exact test was performed for categorical variables, as appropriate. Changes in both GA and LA were analyzed using analysis of variance in a linear mixed model. The chi-square test was used to compare CSF leakage, whereas Fisher exact test was used to compare spinal adhesion, infection, and hematoma.

## 3. Results

### 3.1. Patient demographics

The laminectomy and laminoplasty groups consisted of 59 and 51 patients, respectively. The laminectomy group demonstrated a marginally higher percentage of male individuals (55.93%), whereas the laminoplasty group demonstrated a higher proportion of female individuals (66.67%). No significant differences in body mass index were observed between the groups. In addition, bone densitometry did not indicate any significant differences between the groups, with the mean bone densitometry T-score being ‐2.6 ± 0.95 and ‐2.91 ± 0.96 in the laminectomy and laminoplasty groups, respectively.

We observed no significant differences between the groups regarding medical history, including hypertension, diabetes, rheumatic disease, or thyroid disease. Regarding tumor location, both groups predominantly exhibited intradural extramedullary lesions (*P* < .001). Schwannomas were the most prevalent tumor type in both groups, followed by meningiomas (*P* = .007).

We observed no significant difference in tumor size between the groups. The laminectomy group comprised 13 (22.03%), 31 (52.54%), and 15 (25.42%) tumor cases at the cervical, thoracic, and lumbar levels, respectively. The laminoplasty group comprised 7 (13.72%), 19 (37.25%), and 25 (49.01%) tumor cases at the cervical, thoracic, and lumbar levels, respectively (Table 1). There were 5 cases with spinal tumors in the junctional regions, including 1 and 4 cases of tumors at the T2 and T12 levels, respectively. In the radiographic images, the T2 and T12 levels were included as part of the cervical and lumbar levels, respectively, to recalculate GA.

**Table 1:** Comparison of demographic and clinical data of the laminectomy and laminoplasty groups.

	Laminectomy (N = 59)	Laminoplasty (N = 51)	*P*-value
Age	56.73 ± 14.38	50.61 ± 16.74	.078
Sex (male/female)	33/26	17/34	.029
BMI	23.94 ± 3.74	23.69 ± 3.35	.755
BDM	‐2.6 ± 0.95	‐2.91 ± 0.96	.416
Medical history (%)			.925
Hypertension	20 (33.9)	17 (33.33)	>.999
Diabetes	7 (11.86)	5 (9.8)	.969
Rheumatic disease	0 (0)	2 (3.92)	.213
Thyroid disease	3 (5.08)	9 (17.65)	.072
Antiplatelet (%)	7 (11.86)	5 (9.8)	.969
Pathology (%)			.007
Neurofibroma	5 (8.47)	1 (1.96)	
Schwannoma	20 (33.9)	20 (39.22)	
Lipoma	0	2 (3.92)	
Lymphoma	0	0 (0)	
Astrocytoma	0	2 (3.92)	
Ependymoma	1 (1.69)	3 (5.88)	
Meningioma	11 (18.64)	15 (29.41)	
Chordoma	1 (1.69)	0 (0)	
Hemangioma	4 (6.78)	5 (9.8)	
Others	17 (28.81)	3 (5.88)	
Tumor location (%)			<.001
Intramedullary	5 (8.47)	7 (13.73)	
Intradural extramedullary	32 (54.24)	43 (84.31)	
Extradural	22 (37.29)	1 (1.96)	
Tumor level (%)			.74
Cervical	13 (22.03)	7 (13.72)	
Thoracic	31 (52.54)	19 (37.25)	
Lumbar	15 (25.42)	25 (49.01)	

BDM = bone densitometry, BMI = body mass index.

### 3.2. Radiological assessment

#### 3.2.1. Cervical level

In the laminectomy group, the GA at the cervical level was 10.5 ± 6.82° before surgery, 10.53 ± 5.47° on immediate postoperative, and 9.16 ± 6.5° at the last follow-up. In the laminoplasty group, GA was 8.66 ± 5.06° before surgery, 11.24 ± 8.82° on immediate postoperative, and 15.86 ± 12.54° at the last follow-up. We observed no significant differences in the cervical GA between the groups.

Comparison of GA before and immediately after surgery indicated that kyphosis was induced in the laminoplasty group (*P* = .002). However, it was difficult to conclude whether kyphosis was more pronounced in the laminectomy group than in the final follow-up group. Comparison of the GA before surgery and at the last follow-up within the laminoplasty group demonstrated a trend toward kyphosis progression (*P* = .48). However, the difference was insignificant (*P* = .432).

Regarding the LA before and immediately after surgery, the laminectomy and laminoplasty groups demonstrated a difference of 3.29 ± 9.78° and 2.38 ± 5.98°, respectively. Regarding the LA before surgery and at the last follow-up, the laminectomy and laminoplasty groups demonstrated a difference of 2.55 ± 2.66° and 4.27 ± 19.04°, respectively. We observed no significant differences in LA changes between the groups (Table 2).

**Table 2:** Comparison of the global Cobb angle at the cervical level postoperatively between the laminectomy and laminoplasty groups at the end of follow-up.

Cervical	Laminectomy (N = 13)	Laminoplasty (N = 7)	*P*-value
Pre-op GA (°)	10.5 ± 6.82	8.66 ± 5.06	.605
POD 1 GA (°)	10.53 ± 5.47	11.24 ± 8.82	.895
Last FU GA (°)	9.16 ± 6.5	15.86 ± 12.54	.092
Δ			
(Pre-op) ‐ (POD 1) GA	0.43 ± 4.19	3.12 ± 7.38	.002
(Pre-op) ‐ (Last FU) GA	0.61 ± 5.83	7.20 ± 8.40*	.432
(POD 1) ‐ (Last FU) GA	2.27 ± 5.51	5.06 ± 13.78	.064
Δ			
(Pre-op) ‐ (POD 1) LA	3.29 ± 9.78	2.38 ± 5.98	.328
(Pre-op) ‐ (Last FU) LA	2.55 ± 2.66	4.27 ± 19.04	.223

FU = follow-up, GA = global Cobb angle, LA = local Cobb angle.

* *P*-value = .048.

#### 3.2.2. Thoracic level

In the laminectomy group, the GA at the thoracic level was 29.94 ± 9.56° before surgery, 30.42 ± 11.42° on immediately after surgery, and 28.41 ± 12.58° at the last follow-up. In the laminoplasty group, the GA was 27.89 ± 9.93° preoperatively, 23.58 ± 9.28° immediately after surgery, and 23.18 ± 9.1° at the last follow-up. We observed no significant differences in thoracic GA between the groups.

Upon examining the absolute GA before and immediately after surgery, we observed a trend of kyphosis progression in the laminectomy group compared with in the laminoplasty group (*P* = .011). However, when compared for the last follow-up, there was no significant difference in GA between the groups. Difference in LA indicated a tendency for the LA to increase in the laminectomy group; however, there were no significant differences were observed between the groups (Table 3).

**Table 3:** Comparison of the global Cobb angle postoperatively between the laminectomy and laminoplasty groups at the end of the follow-up at the thoracic level.

Thoracic	Laminectomy (N = 31)	Laminoplasty (N = 19)	*P*-value
Pre-op GA (°)	29.94 ± 9.56	27.89 ± 9.93	.519
POD 1 GA (°)	30.42 ± 11.42	23.58 ± 9.28	.111
Last FU GA (°)	28.41 ± 12.58	23.18 ± 9.10	.542
Δ			
(Pre-op) ‐ (POD 1) GA	9.72 ± 5.48	5.25 ± 3.81	.011
(Pre-op) ‐ (Last FU) GA	8.90 ± 6.70	7.80 ± 4.58	.542
(POD 1) ‐ (Last FU) GA	5.41 ± 5.23	7.46 ± 2.53	.153
Δ			
(Pre-op) ‐ (POD 1) LA	2.89 ± 19.76	1.99 ± 7.17	.199
(Pre-op) ‐ (Last FU) LA	4.09 ± 31.53	2.45 ± 18.02	.141

FU = follow-up, GA = global Cobb angle, LA = local Cobb angle.

#### 3.2.3. Lumbar level

**Figure 3.**
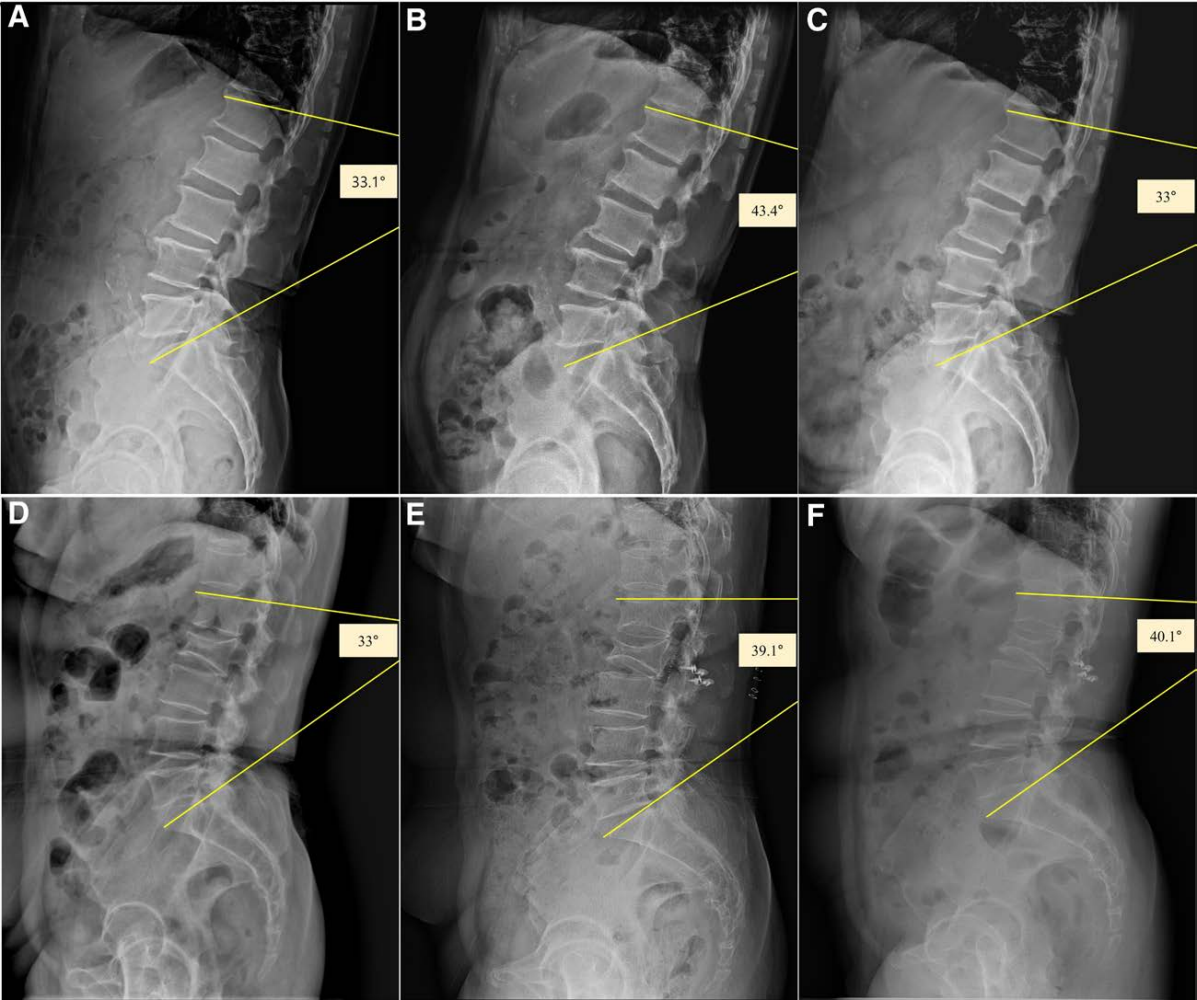
Case-based radiographic comparison: Spinal alignment changes in laminectomy versus laminoplasty. A patient with spinal metastasis originating from prostate cancer at the L2/3 level underwent L2 laminectomy. Comparing the pre-op GA of 33.1° (A), immediate postoperative GA of 43.4° (B), and last follow-up GA of 33° (C), there was no significant increase in spinal instability. Additionally, no complications were reported after laminectomy. Similarly, a patient with a schwannoma at the L2/3 level underwent L2 laminoplasty. Comparing the pre-op GA of 33° (D), immediate postoperative GA of 39.1° (E), and last follow-up GA of 40.1° (F), there was no significant increase in spinal instability. Additionally, no complications were reported after laminoplasty.

In the laminectomy group, the GA at the lumbar level was 43.65 ± 8.47° before surgery, 36.67 ± 12.26° immediately after surgery, and 37.43 ± 13.32° at the last follow-up. In the laminoplasty group, the GA was 40.86 ± 14.12° before surgery, 35.16 ± 12.3° immediately after surgery, and 42.62 ± 10.39° at the last follow-up. We observed no significant differences in lumbar GA between the groups (Fig. 3).

At the lumbar level, the laminoplasty group exhibited progression in the lordosis recovery GA from before to immediately after surgery (*P* = .029) and from immediately after surgery to the last follow-up (*P* = .007). However, between-group comparison did not indicate statistical evidence suggesting that laminoplasty was more advantageous than laminectomy for lordosis recovery. Upon examining the difference in the LA, the laminoplasty group demonstrated a tendency for maintaining lordotic curvature compared with the laminectomy group; however, no significant difference was observed (Table 4).

**Table 4:** Comparison of the global Cobb angle postoperatively between the laminectomy and laminoplasty groups at the end of follow-up at the lumbar level.

Lumbar	Laminectomy (N = 15)	Laminoplasty (N = 25)	*P*-value
Pre-op GA (°)	43.65 ± 8.47	40.86 ± 14.12	.479
POD 1 GA (°)	36.67 ± 12.26	35.16 ± 12.30	.705
Last FU GA (°)	37.43 ± 13.32	42.62 ± 10.39	.422
Δ			
(Pre-op) ‐ (POD 1) GA	10.00 ± 8.32	9.8 ± 7.04	.914
(Pre-op) ‐ (Last FU) GA	8.28 ± 6.24	7.45 ± 7.11	.548
(POD 1) ‐ (Last FU) GA	10.30 ± 6.16	7.91 ± 7.89	.261
Δ			
(Pre-op) ‐ (POD 1) LA	7.70 ± 12.30	5.96 ± 15.81	1.321
(Pre-op) ‐ (Last FU) LA	7.14 ± 15.87	5.65 ± 21.84	.175

FU = follow-up, GA = global Cobb angle, LA = local Cobb angle.

### 3.3. Postoperative complications

Postoperative complications, including CSF leakage, spinal cord adhesion, infection, and hematoma, were observed in 38.98% and 23.53% of the patients in the laminectomy and laminoplasty groups, respectively. Specifically, CSF leakage was observed in 32.2% and 17.65% of patients in the laminectomy and laminoplasty groups, respectively (*P* = .048).

Spinal adhesions were observed in 3.39% and 3.92% of the patients in the laminectomy and laminoplasty groups, respectively. Infection was reported in 1.69% of the patients in the laminectomy group; by contrast, no infections were reported in the laminoplasty group. Hematoma was observed in 5.08% and 1.96% of the patients in the laminectomy and laminoplasty groups, respectively. Notably, all hematoma cases involved patients who were not receiving anticoagulant therapy. All complications, except CSF leakage, did not show significant between the 2 groups (Table 5).

**Table 5:** Comparison of the incidence of postoperative complications between the laminectomy and laminoplasty groups at the end of follow-up.

	Laminectomy (N = 59)	Laminoplasty (N = 51)	*P*-value
CSF leakage (%)	32.2	17.65	.048
Spinal adhesion (%)	2.39	3.92	>.999
Infection (%)	1.69	0	>.999
Hematoma (%)	5.08	1.96	.622

CSF = cerebrospinal fluid.

## 4. Discussion

The surgical approach to laminectomy is necessary for accessing the spinal cord in cases of spinal lesions without subsequent lamina restoration. This procedure entails the removal of the supraspinous and interspinous ligaments as well as excision of the normal lamina, potentially leading to stability concerns.^[6,13]^ Neglecting lamina restoration can lead to various complications, including kyphotic deformity, epidural fibrous tissue adhesions, increased vulnerability to trauma, lesion recurrence, heightened risk during reoperation caused by adhesions with the spinal cord and surrounding muscle tissue, and spinal instability.^[6,14–16]^ Conversely, laminoplasty aids in eliminating bony defects and fostering scar tissue formation, which supplements the functions of the supraspinous and interspinous ligaments.^[2]^

Our findings suggest a tendency for less kyphosis progression in the laminoplasty group than in the laminectomy group, although this difference did not reach statistical significance. When comparing the cervical level between preoperatively and immediately postoperatively, the laminoplasty group showed a tendency toward kyphosis. However, the lack of statistical significance in comparing cervical level between the 2 groups at immediately after surgery and the last follow-up makes it challenging to definitively claim a persistent induction of kyphosis. Additionally, a clear assessment immediately after surgery might have been hindered by postoperative pain. Notably, a systematic review and meta-analysis conducted by Byvaltsev et al^[4]^ that analyzed 6 studies suggested the potential superiority of laminoplasty over laminectomy in preventing postoperative spinal deformities. Their analysis involved 175 and 323 patients who underwent laminoplasty and laminectomy, respectively, of whom 21 and 58 developed spinal deformities, respectively. Overall, these findings suggest a significant increase in kyphosis in the laminectomy group (*P* = .01). Similarly, Montano et al^[16]^ indicated laminectomy as a prognostic factor for spinal deformities. In this study, there were no cases of spinal deformity among 30 patients who underwent laminoplasty. However, patients in the laminectomy group experienced worsened neurological symptoms and deformity aggravation (*P* = .009). Lamina attachment could facilitate maintenance of normal anatomy and potentially reduce the development of kyphosis.

Laminoplasty transcends deformity reduction and exhibits potential advantages in preventing postoperative complications. Our study suggests that laminoplasty has a propensity for reduced complications, such as CSF leakage and hematoma. This procedure diminishes hematoma formation, seemingly by addressing bone defects and mitigating CSF leakage through specific surgical techniques such as isolation of the dural suture site from the drain tube.^[5]^ These observations were consistent with those of previous studies. McGirt et al^[12]^ reported a notable reduction in CSF leakage complications among patients undergoing laminoplasty, which further demonstrates the potential of laminoplasty to minimize adverse events. Moreover, Huang et al^[14]^ focused on patients with cervical myelopathy and highlighted the favorable outcomes associated with laminoplasty. Specifically, their findings suggested an absence of reoperations or severe infections in the laminoplasty cohort. Notably, the laminoplasty cohort exhibited superior neurological outcomes than the laminectomy cohort. Taken together, these findings suggest that laminoplasty may confer additional benefits beyond deformity correction by potentially reducing specific postoperative complications, enhancing patient recovery, and improving neurological outcomes.

This study has some limitations. First, the retrospective study design and reliance on single-center data resulted in a limited data volume. These findings may not entirely represent diverse patient demographics or surgical protocols in other healthcare settings, leading to potential discrepancies. Additional research using a prospective, multicenter approach and enrolling a larger patient cohort could enhance the reliability of the study findings. Future prospective, randomized controlled trials may help determine the optimal surgical approach for specific patient subgroups. Second, the use of the “Surgi-map” software ensured accurate and consistent Cobb angle measurements; however, the retrospective study nature and challenges in data extraction from medical records might have introduced inherent selection bias. Despite efforts to maintain precision, relying on a single observer to evaluate spinal alignment with the “Surgi-map” software may have introduced measurement errors. Third, patient selection for surgical procedures lacked standardized criteria and was largely determined by the preference of the operating surgeons, which may have resulted in bias. To address these concerns, we acknowledge the inherent limitations associated with decision-making driven by surgeon preference and the potential for measurement bias. In future research, efforts will be made to establish standardized criteria for surgical procedure selection and to minimize measurement bias through rigorous data collection protocols. Fourth, upon examining the normality assumption of the linear mixed model, we found that our data did not follow a normal distribution. However, we did not identify any suitable alternative statistical methods. While we considered using generalized estimating equations, we concluded that it would not significantly alter the interpretation of our results. Fifth, the study did not reflect the diversity of laminoplasty surgical techniques. However, the study focused on the structural stability and prevention of complications following lamina reconstruction between laminoplasty and laminectomy. Rather than focusing on the differences among various surgical techniques within laminoplasty, we aimed to explore the fundamental advantages of laminoplasty. Since data accumulated starting from 1994 may encompass various techniques in the laminoplasty group, it is plausible that there were advances in surgical techniques during the study period. Therefore, further follow-up studies focusing on recent laminoplasty techniques are warranted. Furthermore, variability in postoperative follow-up schedules might have influenced the assessment of long-term outcomes. Employing a standardized follow-up schedule for all patients could provide more precise and comparable data.

## 5. Conclusions

Our study provides valuable insights into the surgical management of spinal cord tumors. Both laminectomy and laminoplasty are effective options for tumor resection and spinal cord decompression. Nonetheless, laminoplasty demonstrated advantages in reducing postoperative CSF leakage.

By emphasizing the efficacy and safety profile of laminoplasty in spinal cord tumor surgery, we provide valuable insights into ongoing discussions regarding optimal surgical strategies for these patients. Finally, our findings may guide clinical decision-making and improve patient outcomes in this field.

## Author contributions

**Conceptualization:** Pyung Goo Cho.

**Data curation:** Hyun Jin Yoo.

**Formal analysis:** Hyun Jin Yoo.

**Investigation:** Hyun Jin Yoo.

**Supervision:** Sung Hyun Noh, Sang Hyun Kim, Pyung Goo Cho.

**Visualization:** Hyun Jin Yoo.

**Writing – original draft:** Hyun Jin Yoo.

**Writing – review & editing:** Pyung Goo Cho.
